# Supplementary material for: Abyssal hydrothermal alteration drives the evolution from simple alkanes to prebiotic molecular complexity
Source: Nat Commun. 2026 Feb 5;17:2415. doi: 10.1038/s41467-026-68745-1 (PMC12988106; doi:10.1038/s41467-026-68745-1)
Supplement: Supplementary file 1 — Supplementary Information [file 41467_2026_68745_MOESM1_ESM.pdf]

Supplementary Information for

"Abyssal hydrothermal alteration drives the evolution from simple alkanes to prebiotic molecular complexity"

Quanyou Liu[1*], **Huiyuan Xu**[1,2,3*], Jiuyuan Wang[1], Dongya Zhu[2], Chi Zhang[3], Biqing Zhu[1], Yin Fu[3], Wang Zhang[3], Jiangtao Li[4], Di Zhu[5], Shili Liao[6], Chunhui Tao[6], Guanghui Yuan[7], Shang Xu[7], Huaiwei Ni[8], Fang Hao[7], Zhijun Jin[1]

[1] Institute of Energy, School of Earth and Space Sciences, Peking University, Beijing 100871, China

[2] Petroleum Exploration & Production Research Institute, Beijing 100083, China

[3] Institute of Geology and Geophysics, Chinese Academy of Sciences, Beijing 100029, China

[4] School of Ocean and Earth Science, Tongji University, Shanghai 200092, China

[5] School of Sustainable Energy and Resources, Nanjing University, Suzhou 215163, China

[6] State Key Laboratory of Submarine Geoscience, Second Institute of Oceanography, Ministry of Natural Resources, 310012 Hangzhou, China

[7] School of Geosciences, China University of Petroleum (East China), Qingdao 266580, China

[8] School of Earth and Space Sciences, University of Science and Technology of China, Hefei 230026, China

Corresponding authors: liuqy@pku.edu.cn (Q.L.); xuhuiyuan.ian@hotmail.com (H.X.)

Supplementary notes

Validity and advantages of network-informed approaches in hydrothermal organic geochemistry

While network-informed approaches were originally developed for biological systems, the fundamental principles of network-based molecular analysis are universally applicable to any system exhibiting systematic molecular transformations. The adaptation to geochemical systems requires careful consideration of non-enzymatic reaction mechanisms, but offers unprecedented insights into molecular evolution pathways.

The validity of applying metabolomics-inspired approaches to geochemical systems is supported by fundamental similarities: both systems exhibit (1) dynamic molecular networks with reversible transformations, (2) environmental responsiveness that creates systematic molecular patterns, and (3) emergent properties arising from molecular interactions.

This organic geochemical framework offers several advantages over traditional approaches: (1) it captures the full molecular complexity rather than focusing on individual molecular markers, (2) it reveals system-level transformation networks rather than isolated reactions, and (3) it provides predictive power for understanding molecular evolution under extreme conditions.

Mantle origin of hydrothermal vents constrained by Hg isotope compositions

Mercury isotope, as a robust proxy[1,2], is used to determine the mantle origin of the analyzed vent samples. Hg concentrations are mainly in the ppb level in the background intervals. Total Hg concentrations and isotopic compositions of the samples are given in Supplementary Table 1. The overall Hg concentrations range from 8.5 to 13390 ppb with an average of 2445 ppb, and with limited variations in δ^{202}Hg (−1.55‰ to −0.34‰), Δ^{199}Hg (0.0‰ to −0.07‰ with a median of 0.04‰) values and Δ^{201}Hg (−0.02‰ to −0.06‰ with a median of 0.01‰) values (Supplementary Fig. 4). This isotopic fingerprinting suggests limited contribution from marine sedimentary or terrestrial reservoirs, instead pointing to a deep magmatic origin for the Hg in these samples, thus indicates that the analyzed vent samples are of predominantly mantle-derived. The clustering of data points implies a consistent mantle source for all vents, and suggests genetic relationships and synchronized vent fluid conditions among the analyzed materials, providing compelling evidence that deep mantle upwelling is the principal driver for vent conduit formation.

Moreover, no statistically significant correlations were observed between organic proxies and the δ^{202}Hg, Δ^{199}Hg, Δ^{200}Hg and Δ^{201}Hg values. Hg concentrations, TOC and temperature show limited variations and correlations across the locations (Supplementary Fig. 5). Therefore, organic compounds record more distinctive molecular signatures, serving as important markers for revealing the processes within hydrothermal systems at this mantle-seafloor interface.

Supplementary geology

The Indian Ridge, extending approximately 8,000 km between the African and Antarctic plates, represents a significant ultraslow-spreading ridge system (14-16 mm/yr) that connects the Rodriguez Triple Junction (RTJ) to the east and the Bouvet Triple Junction to the west. This study focuses on hydrothermal fields from two distinct tectonic settings: the Longqi field along the Indian Ridge and the Edmond and Kairei fields near the RTJ.

The central section of the Indian Ridge, bounded by the Indomed and Gallieni transform faults, exhibits intensive hydrothermal activity with a site frequency of ~2.5 sites per 100 km, comparable to that of the Mid-Atlantic Ridge at 36°N-38°N. The crustal thickness along the Indian Ridge (approximately 4 km) is notably thinner than adjacent regions, with geophysical studies indicating enhanced magmatic activity since 8-10 Ma. The distribution of hydrothermal activity is primarily controlled by local magma supply and crustal permeability.

The Longqi hydrothermal field (49.65°E, 37.78°S) is situated at water depths between 2900 and 2700 m on the southeast wall of the axial valley. It is characterized by an NW-SE trending bathymetric high and bounded by a prominent N-S trending fault with a ~120 m wide deformation zone. The Longqi hydrothermal field is characterized by mostly active chimney structures. Active venting in the Longqi field exhibits fluid temperatures ranging from 308°C to 358°C. The basement geology consists primarily of extrusive units, including sheet flows, tubes, and pillow basalts.

Near the Rodriguez Triple Junction, the Edmond (69.60°E, 23.88°S) and Kairei (70.04°E, 25.32°S) hydrothermal fields represent distinct evolutionary stages of hydrothermal activity. The Edmond field is dominated by inactive chimneys, while the Kairei field displays both active and inactive chimneys.

Supplementary codes

Detailed analysis scripts are provided below:

```
import pandas as pd
import numpy as np
from Bio.Phylo.TreeConstruction import DistanceMatrix, DistanceTreeConstructor

def upgma_tree(path,name,unknown=True):
    '''
    In the path, fill in the file path with 'r'.
    Enter the file name in name
    unknown=False only excludes siloxanes
    unknown=True excludes siloxanes and unknowns
    '''
    path_name = path + '\\' + name
    data = pd.read_excel(path_name,sheet_name='相似度矩阵 Similarity Matrix ')
    data_name = pd.read_excel(path_name)
    if unknown:
        data_name = data_name.loc[:,'化合物编号 Alignment
ID'].dropna(how='any').drop(index=data_name[data_name.loc[:,'化合物名称
Metabolite name'].str.contains('siloxane|Unknown',na=False)].index)
    else:
        data_name = data_name.loc[:, '化合物编号 Alignment
```

```
ID'].dropna(how='any').drop(index=data_name[data_name.loc[:,'化合物名称
Metabolite name'].str.contains('siloxane', na=False)].index)
    data = data[data['SourceID'].isin(data_name.astype(int)) &
data['TargetID'].isin(data_name.astype(int))]

    # Extract all unique compound IDs
    all_ids = sorted(set(data['SourceID']).union(set(data['TargetID'])))
    n = len(all_ids)
    id_to_idx = {id: idx for idx, id in enumerate(all_ids)}

    # Initialize the distance matrix
    dist_matrix = np.ones((n, n))
    np.fill_diagonal(dist_matrix, 0)

    # Fill in the known distance
    for _, row in data.iterrows():
        i = id_to_idx[row['SourceID']]
        j = id_to_idx[row['TargetID']]
        dist = 1 - row['Score']
        dist_matrix[i, j] = dist
        dist_matrix[j, i] = dist
```

```
    # Convert the distance matrix to lower triangular format
    n = len(all_ids)
    dm_list = []
    for i in range(n):
        dm_list.append(dist_matrix[i, :i+1].tolist())

    # Create a DistanceMatrix object
    labels = [f"{str(id)}" for id in all_ids]
    dm = DistanceMatrix(names=labels, matrix=dm_list)

    # Constructing a tree using the UPGMA method
    constructor = DistanceTreeConstructor()
    upgma_tree = constructor.nj(dm)

    # Save as a Newick format file
    newick_str = upgma_tree.format('newick')
    with open(f"upgma_tree({name})(nj).newick", "w") as f:
        f.write(newick_str)

upgma_tree(path=r'path',name='Aliphatic data refined 0%.xlsx',unknown=True)
```

Supplementary tables

Supplementary Table 1 The information of locations, activities, measured temperature, lowest pH (measured at 25°C and 1 atm), internal minerals, coordinates and bulk organic geochemical characteristics (Rock mass used, total organic carbon content and total liquid extracts) of hydrothermal vent samples from Longqi, Edmond, and Kairei hydrothermal fields along the Indian Ridge.

Region	Activity	Sample codes	Temperature at vents/℃	Lowest pH (measured at 25°C and 1 atm)	Internal minerals	Sampling coordinates	Rock/g	TOC/wt%	TLE/wt%
Longqi	Active	SY111-G10			Chalcopyrite	49.64751°E, 37.78100°S	49.09	0.073	0.28
	Active	SY097-G01	High (~300)		Chalcopyrite	49.64768°E, 37.78049°S			
	Active	SY105	High (358-362)	3.32	Consists of compact chalcopyrite, with white anhydrite in the center	49.64908°E, 37.78371°S	48.09	0.076	0.26
	Active	SY104-G06			Chalcopyrite and white anhydrite	49.64917°E, 37.78364°S			
	Active	SY109-G07	308		Abundant chalcopyrite	49.64885°E, 37.78271°S	15.48	0.054	0.92
	Inactive	SY093-G07			Low chalcopyrite content	49.64909°E, 37.78394°S	40.29	0.064	0.02
	Active	SY107	High (358)	4.85	Interior composed of chalcopyrite	49.64985°E, 37.78371°S	23.83	0.052	0.01
Edmond	Inactive	SY139-G7			Reddish-brown oxides with sea anemones attached	69.5963°E, 23.8783°S	55.68	0.068	0.28
	Inactive	SY134-G06			Interior consists of black sulfides	69.59651°E, 23.87844°S			
	Inactive	SY150-G6			Covered with yellowish altered sulfides	69.596766°E, 23.878050°S	16.17	0.295	0.03
Kairei	Active	SY147-G8				70.04013°E, 25.32041°S	21.3	0.052	0.01
	Inactive	SY145-G1			Conduit is filled with sulfides, chalcopyrite	70.04022°E, 25.32032°S	28.11	0.061	0.01

Supplementary Table 2 Geochemical characteristics of mercury concentrations, Hg isotope compositions of hydrothermal vent samples from Longqi, Edmond, and Kairei hydrothermal fields along the Indian Ridge.

Region	Activity	Sample codes	Hg （ppb）	δ^{199}Hg (‰)	2SD	δ^{200}Hg (‰)	2SD	δ^{201}Hg (‰)	2SD	δ^{202}Hg (‰)	2SD	Δ^{199}Hg (‰)	2SD	Δ^{200}Hg (‰)	2SD	Δ^{201}Hg (‰)	2SD
Longqi	Active	SY111-G10	103.2	-0.077	0.070	-0.255	0.038	-0.370	0.056	-0.492	0.071	0.047	0.067	-0.008	0.031	0.000	0.043
	Active	SY097-G01															
	Active	SY105	963.4	-0.136	0.070	-0.388	0.038	-0.561	0.056	-0.786	0.071	0.062	0.067	0.007	0.031	0.030	0.043
	Active	SY104-G06															
	Active	SY109-G07															
	Inactive	SY093-G07	8.5	-0.349	0.070	-0.751	0.038	-1.108	0.056	-1.550	0.071	0.042	0.067	0.028	0.031	0.057	0.043
	Active	SY107	1841.0	-0.115	0.070	-0.317	0.038	-0.493	0.056	-0.656	0.071	0.051	0.067	0.013	0.031	0.001	0.043
Edmond	Inactive	SY139-G7	473.8	-0.201	0.070	-0.391	0.038	-0.573	0.056	-0.788	0.071	-0.002	0.067	0.005	0.031	0.020	0.043
	Inactive	SY134-G06	13390.0	-0.164	0.070	-0.454	0.038	-0.652	0.056	-0.945	0.071	0.074	0.067	0.021	0.031	0.058	0.043
	Inactive	SY150-G6															
Kairei	Active	SY147-G8	316.7	-0.137	0.070	-0.250	0.038	-0.435	0.056	-0.579	0.071	0.009	0.067	0.041	0.031	0.000	0.043
	Inactive	SY145-G1	2460.0	-0.084	0.070	-0.198	0.038	-0.272	0.056	-0.335	0.071	0.000	0.067	-0.029	0.031	-0.020	0.043

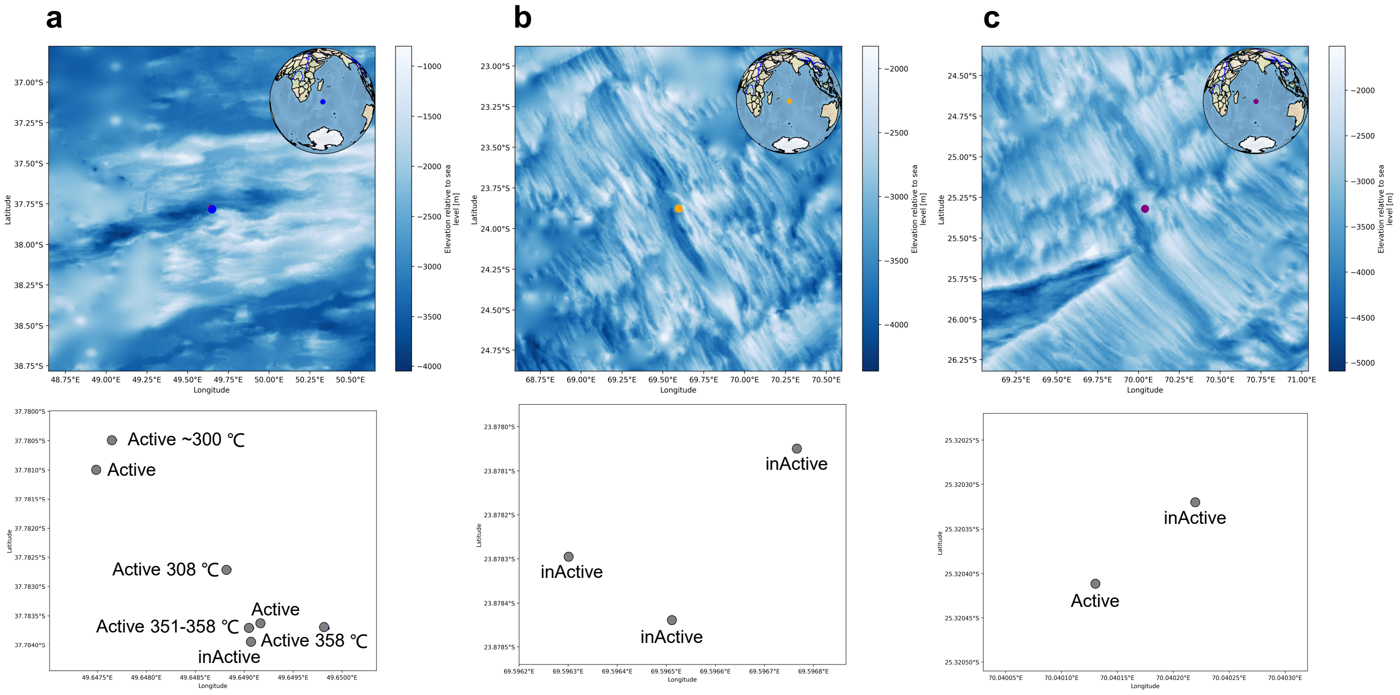

Supplementary Fig. 1 **Bathymetric context and hydrothermal characteristics of sampling stations across three Indian Ocean vent fields**. Spatial distribution, activity state, and representative vent-fluid temperatures of sampling stations from the Longqi (**a**), Edmond (**b**), and Kairei (**c**) hydrothermal fields on the Indian Ridge, indicated respectively by blue, orange, and magenta markers. Upper panels display that shaded-relief bathymetry is shown for Longqi (centered at 49.6475° E, 37.7810° S), Edmond (69.5963° E, 23.8783° S), and Kairei (70.0401° E, 25.3204° S), indicating the regional tectonic context of each vent field along its respective mid-ocean ridge segment. Color scaling from light grey to dark blue denotes seafloor elevation from –1,000 m to –5,000 m below mean sea level. Insets highlight the position of each field along the ultraslow- to slow-spreading Indian Ridge. Bottom panels display detailed coordinate plots of sampled sites annotated with vent activity status (Active vs. Inactive) and, where available, vent fluid temperatures. Bathymetric data were obtained from the GEBCO 2024 gridded dataset, which provides global, publicly available seafloor elevation data (https://www.gebco.net/data-products/gridded-bathymetry-data; GEBCO Compilation Group (2025) GEBCO 2025 Grid (doi: 10.5285/37c52e96-24ea-67ce-e063-7086abc05f29)). The software used is open-source and publicly available.

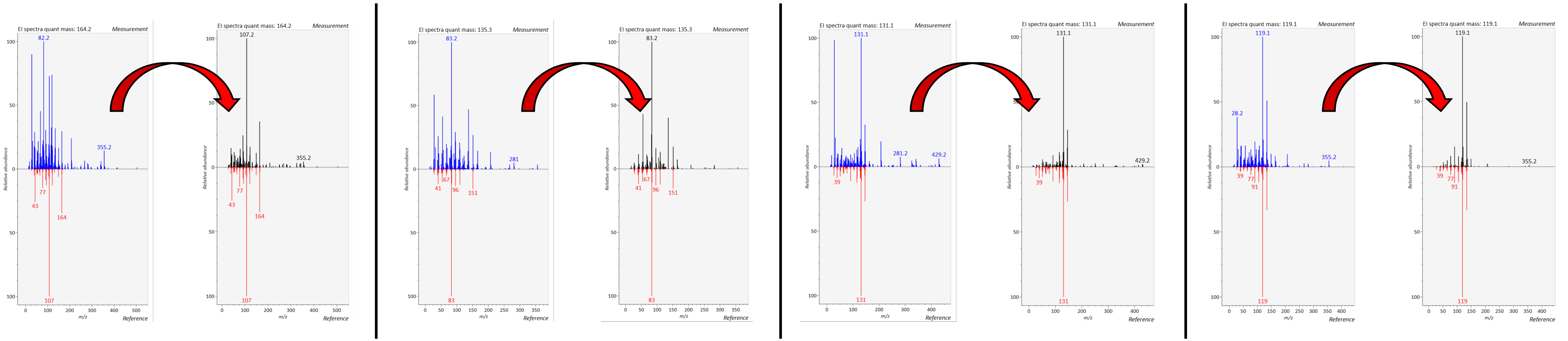

2-Butanone, 4-(4-hydroxyphenyl)- 1-Cyclopentyl-1H-pyrazol-5-amine 1H-Indene, 2,3-dihydro-1,6-dimethyl- Benzene, 2-ethyl-1,4-dimethyl-

Supplementary Fig. 2 **Demonstration of the deconvolution of mass spectra of organic compounds**. Red arrows indicate algorithmic purification processes. Blue are untreated mass spectra.

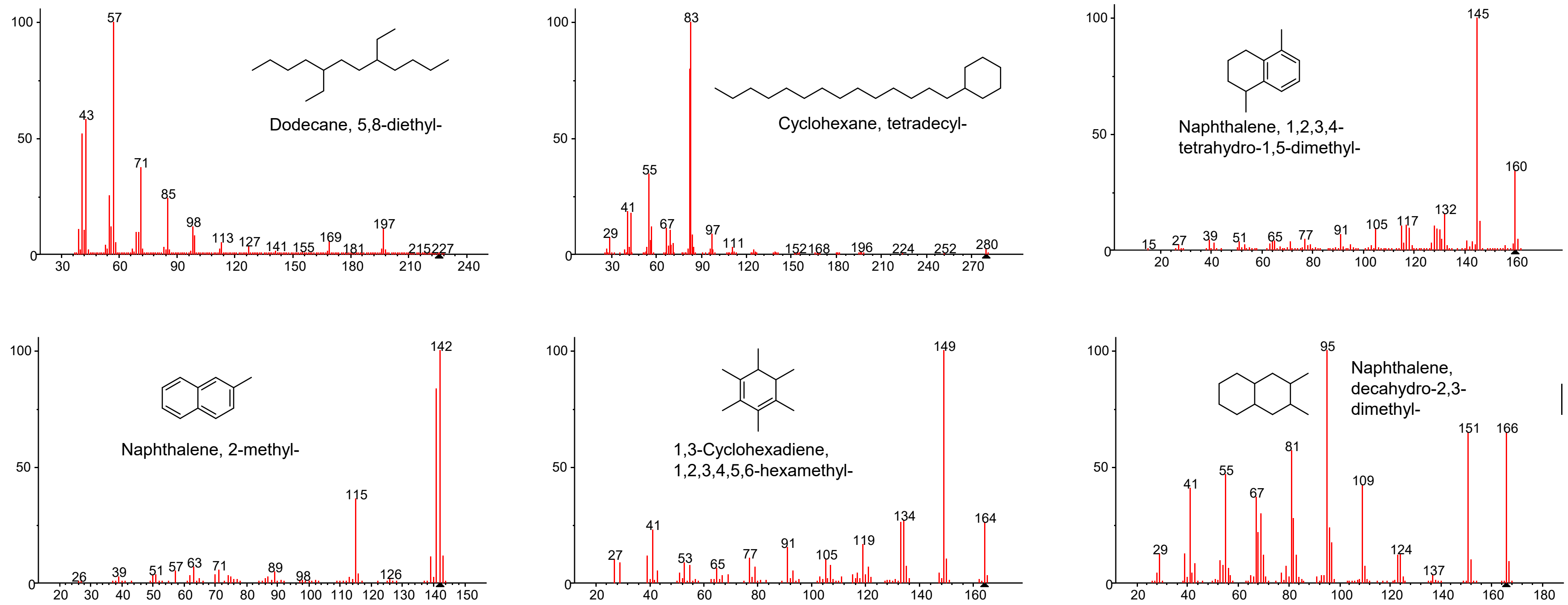

Supplementary Fig. 3 **Mass spectra of representative organic compounds co-detected and co-annotated in vent samples**.

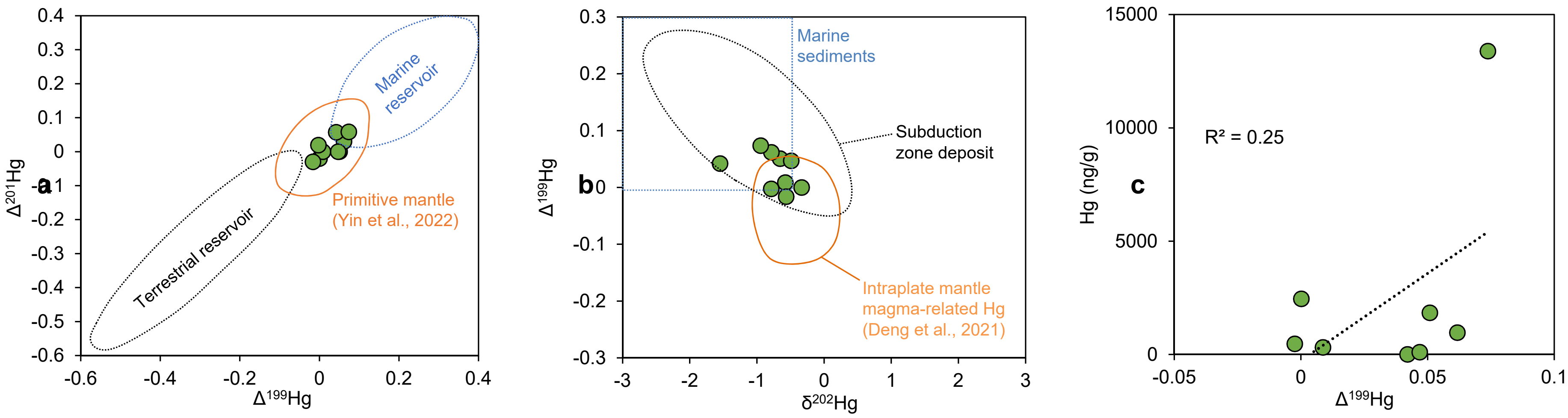

Supplementary Fig. 4 **Mercury isotopic compositions reveal dominant mantle-derived source in hydrothermal samples.** Cross-plots showing Hg abundance and Hg isotopes for all analysed samples and their implications for mercury provenance. ***a****, mass-independent fractionation* Δ^{199}*Hg versus* Δ^{201}*Hg* ***b,*** *mass-dependent (*δ^{202}*Hg) versus mass-independent fractionation (*Δ^{199}*Hg);* ***c*,***. Hg concentration versus mass-independent fractionation* Δ^{199}*Hg*. Reference isotopic fields are outlined for comparison: the terrestrial crustal reservoir (black dotted ellipse), marine sedimentary reservoir (blue dashed ellipse), the primitive mantle array reported by **Yin, et al.** [1] (orange ellipse in ***a***), and intraplate-mantle magma–related Hg characterised by **Deng, et al.** [2] (orange ellipse in ***b***). The grey dotted outline in ***b*** represents subduction-zone Hg deposits. Sample clusters overlap the primitive- to intraplate-mantle fields and plot distinctly away from the marine sedimentary and crustal reservoirs, signifying a dominant mantle-derived Hg component with negligible sedimentary contribution.

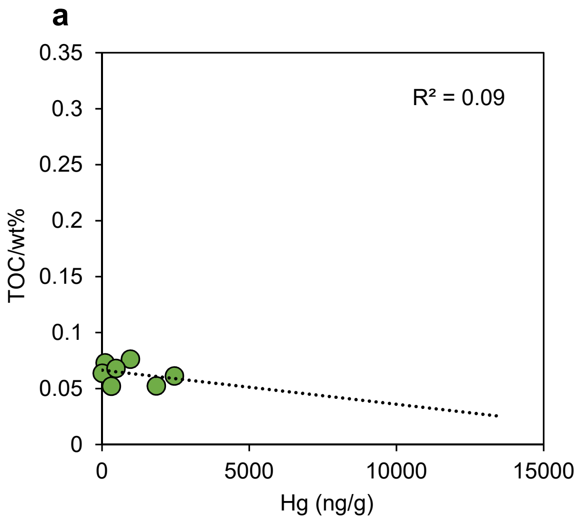

Supplementary Fig. 5 **Lack of correlation between mercury content, isotopes, and organic carbon in hydrothermal samples. Cross-plots showing relationships between mercury concentrations, organic matter, and mercury isotopes in the studied samples. a,** Cross-plot between total organic carbon (TOC, wt%) and mercury concentrations (Hg, ng/g) showing negligible correlation (R^2 = 0.09), suggesting limited influence of organic matter on mercury sequestration. **b,** Cross-plot of total liquid extract (TLE, wt%) versus δ^{201}Hg values (‰), displaying no correlation (R^2 = 0.02), which indicates that the isotopic signature of mercury is not controlled by total liquid extracts. **c,** Cross-plot of TOC (wt%) versus δ^{201}Hg values (‰) exhibiting essentially no correlation (R^2 = 0.01), further implying that mercury sources and isotopic fractionation processes are largely independent of organic carbon variation.

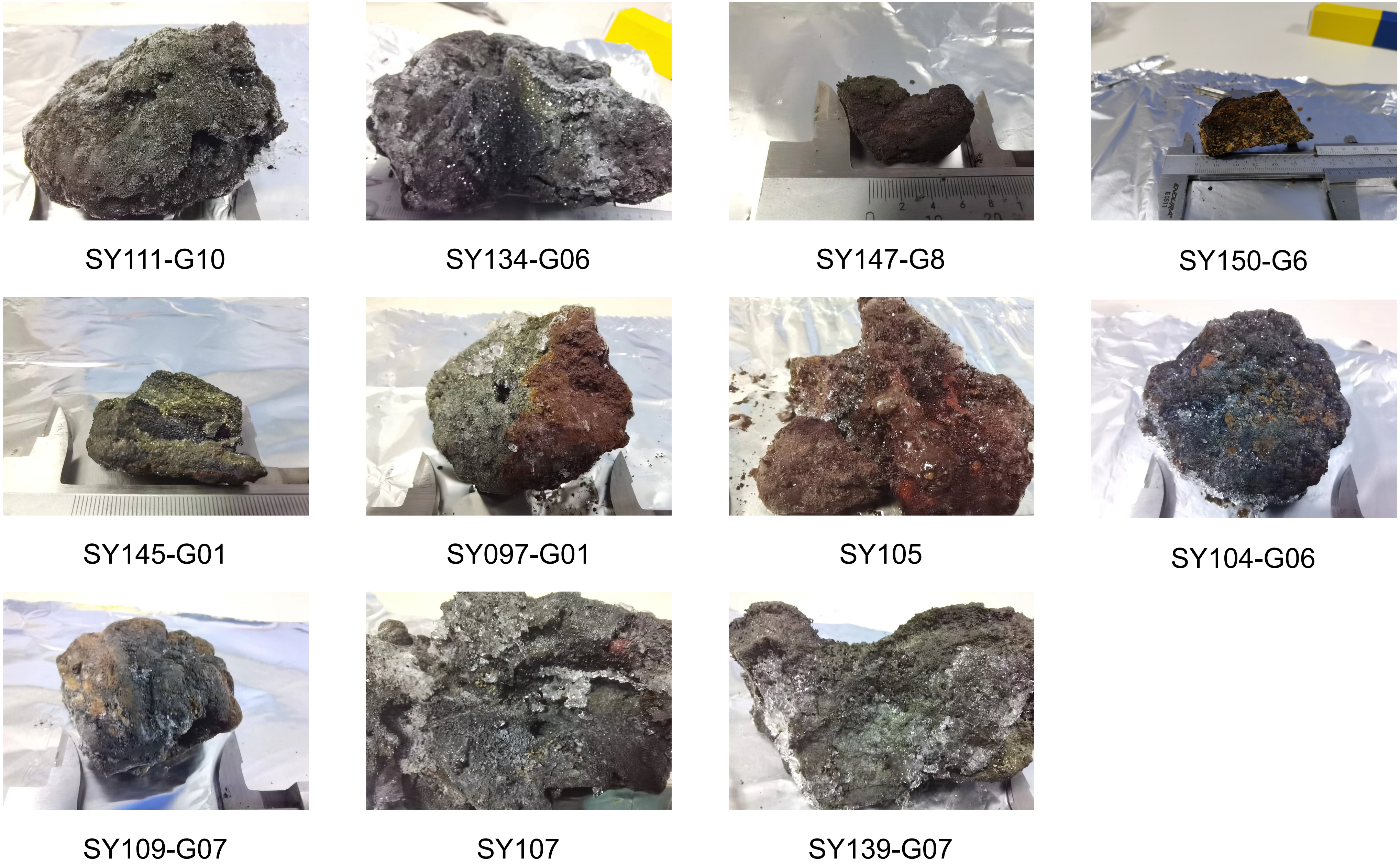

Supplementary Fig. 6 **Photographs of original chimney samples collected from Indian Ridge hydrothermal vent fields**.

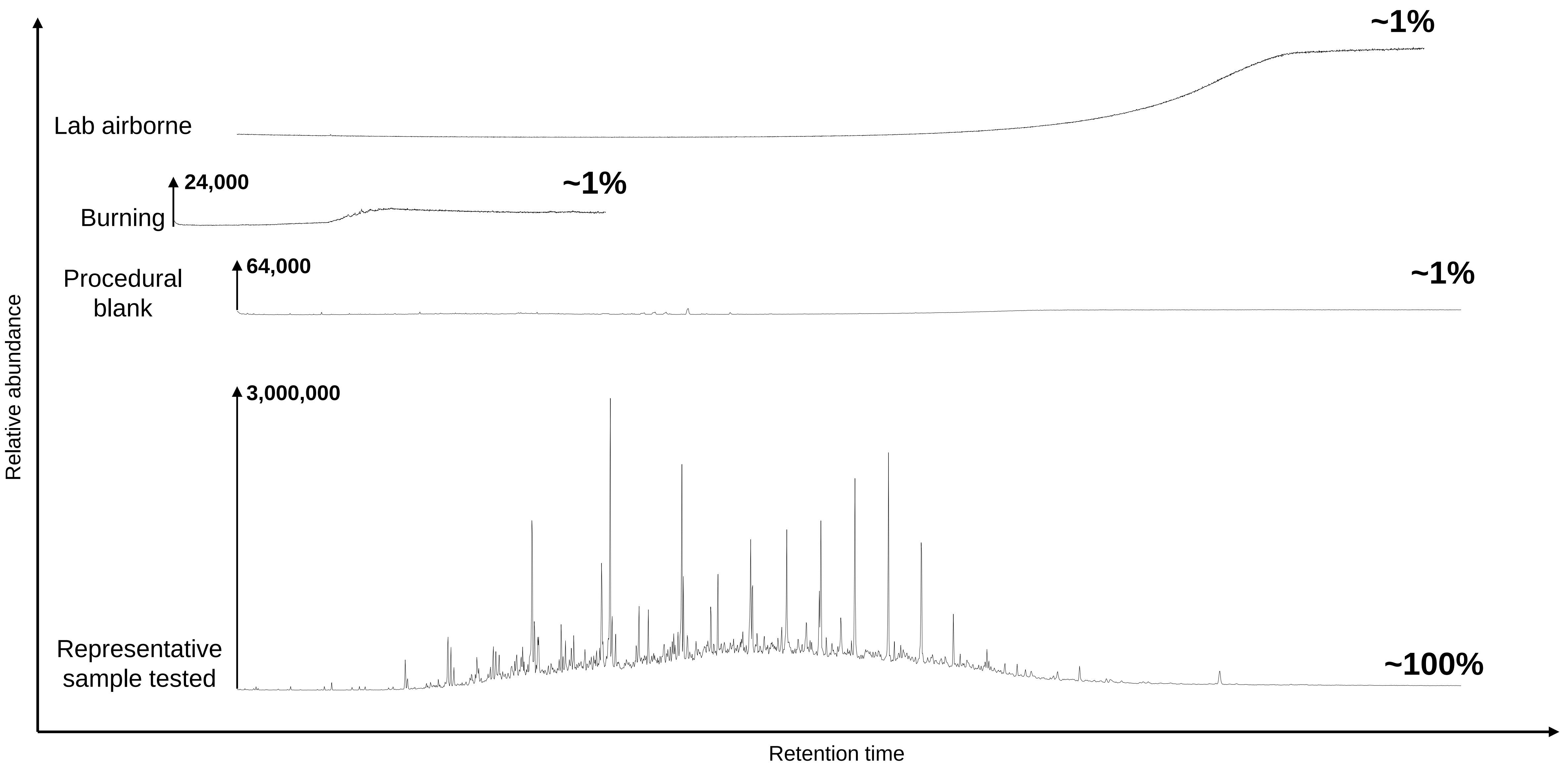

Supplementary Fig. 7 **Mass chromatograms demonstrate null contamination levels in soluble organic extracts.** Mass chromatograms showing total ion current (TIC) of a representative chimney sample extract (bottom) compared with procedural controls, including procedural blank, burning blank, and lab airborne blank (top three traces). The y-axis indicates relative abundance, with the sample normalized to 100% (~3,000,000), while all blanks remain below ~1% relative intensity. Specifically, airborne background (~24,000), combustion (~64,000), and procedural blank signals show minimal ion presence and no major overlapping peaks with the sample. This confirms that lab airborne contamination, combustion-derived interference, and procedural blank contributions are negligible. The chromatographic contrast highlights the effectiveness of our laboratory protocol in minimizing external contamination during extraction and analysis. All controls show flat baselines with no diagnostic peaks resembling the sample's abundant and diverse compound distribution. These results ensure the robustness and authenticity of the detected hydrothermal molecular signals.

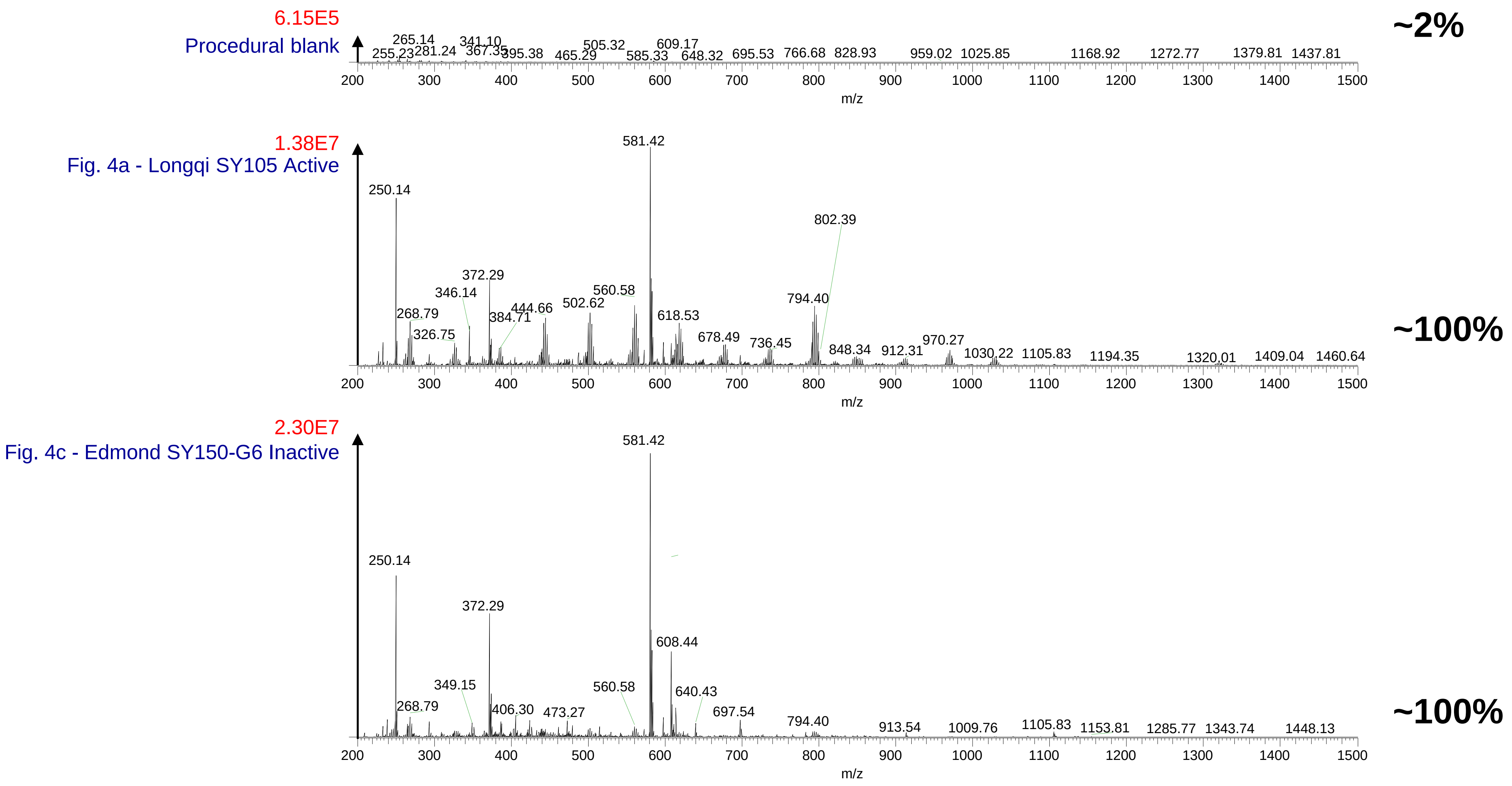

Supplementary Fig. 8 **Fourier transform mass spectra confirm negligible laboratory background in hydrothermal chimney extracts.** Fourier transform mass spectra (FT-MS) comparing the procedural blank (top panel) with two representative chimney samples from different venting states: Longqi SY105 Active (middle) and Edmond SY150-G6 Inactive (bottom). All spectra are plotted across m/z 200–1500. The procedural blank shows low-intensity signals (~2% of the sample signal), with no dominant peaks overlapping with the two chimney spectra. In contrast, the sample spectra exhibit rich and abundant molecular ion signals spanning a wide m/z range, with peak intensities over 100× greater than those in the blank. These results demonstrate that external laboratory contamination does not significantly influence the detected mass spectral features and support the indigenous origin of detected compounds in chimney extracts.

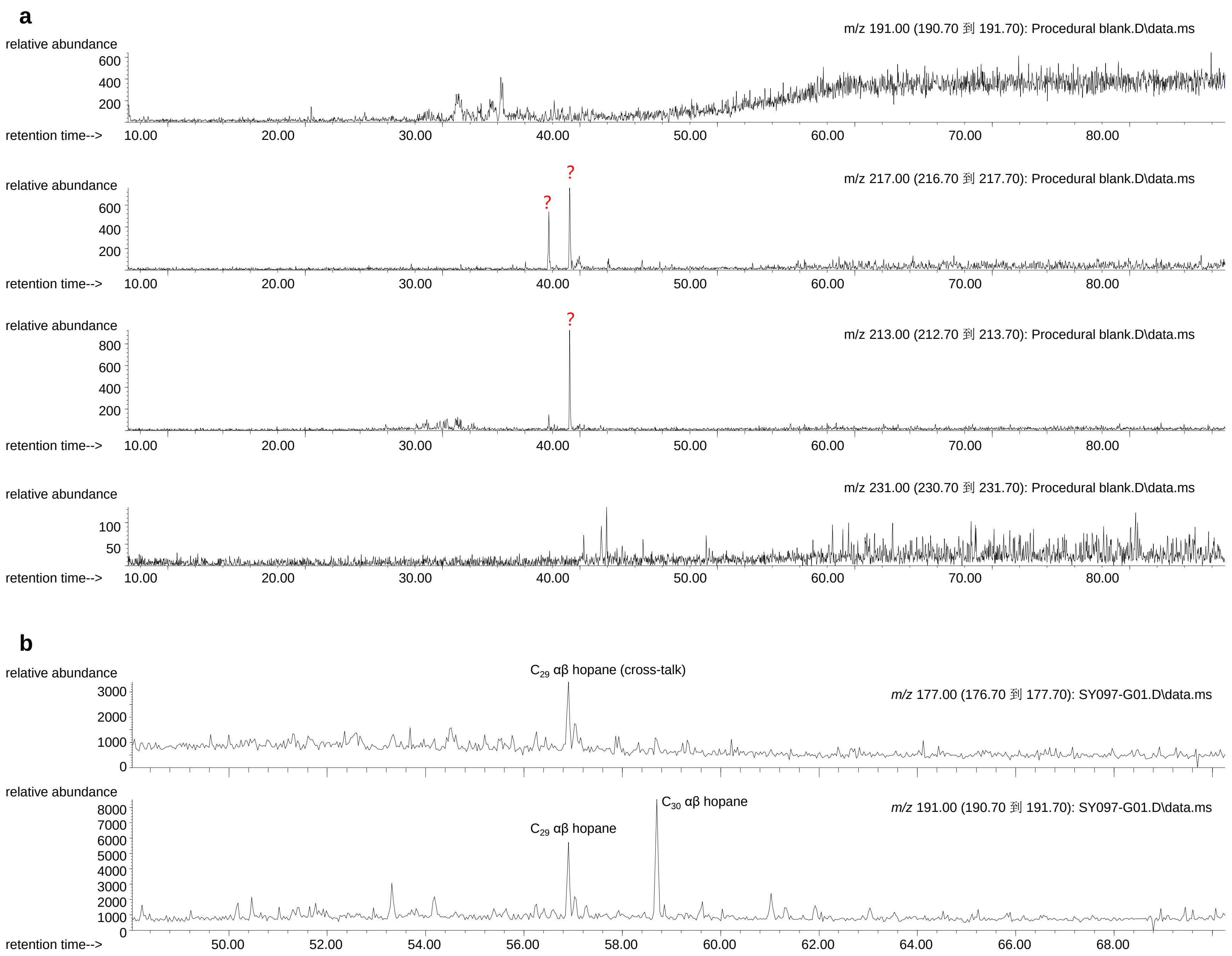

Supplementary Fig. 9 **Mass chromatographic evidence for null sterol contamination and absence of secondary microbial biomarkers. a,** Mass chromatograms of *m/z* 191, 217, 213 and 231 from procedural blank samples demonstrate negligible background sterol contamination during sample workup. Across all traces, signal intensities remain low, and sterol-related peaks are absent, supporting the cleanliness of laboratory protocols and excluding exogenous contamination as a source of sterol-related features in chimney samples. **b,** Mass chromatograms of *m/z* 177 and 191 of a representative chimney extract (sample SY097-G01) show clear C_{30} αβ hopane signals but no evidence for C_{29} norhopanes, suggesting minimal to no microbial biodegradation. The absence of norhopanes excludes extensive post-depositional microbial alteration of the preserved organic matter. The hopane signals indicate the thermal maturity is largely driven by abiotic, hydrothermal processes rather than biological alteration.

Supplementary references

1 Yin, R. *et al.* Mantle Hg isotopic heterogeneity and evidence of oceanic Hg recycling into the mantle. *Nat. Commun.* **13,** 948 (2022).

2 Deng, C. *et al.* Recycling of mercury from the atmosphere-ocean system into volcanic-arc–associated epithermal gold systems. *Geology* **49,** 309-313 (2021).
