# Supplementary material for: Abyssal hydrothermal alteration drives the evolution from simple alkanes to prebiotic molecular complexity
Source: Nat Commun. 2026 Feb 5;17:2415. doi: 10.1038/s41467-026-68745-1 (PMC12988106; doi:10.1038/s41467-026-68745-1)
Supplement: Supplementary file 2 — Description of Additional Supplementary Files [file 41467_2026_68745_MOESM2_ESM.pdf]

## Description of Additional Supplementary Files:

**Supplementary Data 1:** The molecular relatedness tree file. The output based on similarity among co-annotated mass spectra across all samples.

**Supplementary Movie 1:** Partial summed 3D mass spectra of representative vent extracts showing the mass ranges and retention times obtained by hard ionization gas chromatography-mass spectrometry. Colour denotes general chemical families used in this study
