# Supplementary material for: Abyssal hydrothermal alteration drives the evolution from simple alkanes to prebiotic molecular complexity
Source: Nat Commun. 2026 Feb 5;17:2415. doi: 10.1038/s41467-026-68745-1 (PMC12988106; doi:10.1038/s41467-026-68745-1)
Supplement: Supplementary file 5 — Reporting Summary [file 41467_2026_68745_MOESM5_ESM.pdf]

nature portfolio

Corresponding author(s): Quanyou Liu, Huiyuan Xu

Last updated by author(s): Oct 20, 2025

Reporting Summary

Nature Portfolio wishes to improve the reproducibility of the work that we publish. This form provides structure for consistency and transparency in reporting. For further information on Nature Portfolio policies, see our Editorial Policies and the Editorial Policy Checklist.

Statistics

For all statistical analyses, confirm that the following items are present in the figure legend, table legend, main text, or Methods section.

n/a	Confirmed	
x		The exact sample size (*n*) for each experimental group/condition, given as a discrete number and unit of measurement
	x	A statement on whether measurements were taken from distinct samples or whether the same sample was measured repeatedly
x		The statistical test(s) used AND whether they are one- or two-sided<br>*Only common tests should be described solely by name; describe more complex techniques in the Methods section.*
x		A description of all covariates tested
x		A description of any assumptions or corrections, such as tests of normality and adjustment for multiple comparisons
x		A full description of the statistical parameters including central tendency (e.g. means) or other basic estimates (e.g. regression coefficient) AND variation (e.g. standard deviation) or associated estimates of uncertainty (e.g. confidence intervals)
x		For null hypothesis testing, the test statistic (e.g. *F*, *t*, *r*) with confidence intervals, effect sizes, degrees of freedom and *P* value noted<br>*Give P values as exact values whenever suitable.*
x		For Bayesian analysis, information on the choice of priors and Markov chain Monte Carlo settings
x		For hierarchical and complex designs, identification of the appropriate level for tests and full reporting of outcomes
x		Estimates of effect sizes (e.g. Cohen's *d*, Pearson's *r*), indicating how they were calculated

Our web collection on statistics for biologists contains articles on many of the points above.

Software and code

Policy information about availability of computer code

Data collection	Agilent Chemstation, Thermo Fisher Xcaliber, MSdial
Data analysis	Mass spectral data comparison was performed using an open-source software from https://systemsomicslab.github.io/compms/msdial/main.html. Tree was generated using a free tool of iTol. Python scripts (in SI) was used for data matrix construction

For manuscripts utilizing custom algorithms or software that are central to the research but not yet described in published literature, software must be made available to editors and reviewers. We strongly encourage code deposition in a community repository (e.g. GitHub). See the Nature Portfolio guidelines for submitting code & software for further information.

Data

Policy information about availability of data

All manuscripts must include a data availability statement. This statement should provide the following information, where applicable:

- Accession codes, unique identifiers, or web links for publicly available datasets
- A description of any restrictions on data availability
- For clinical datasets or third party data, please ensure that the statement adheres to our policy

Date are presented in the Paper or in Supplementary Information, and raw data files have been deposited in the Zenodo database at https://doi.org/10.5281/zenodo.17398264

Research involving human participants, their data, or biological material

Policy information about studies with human participants or human data. See also policy information about sex, gender (identity/presentation), and sexual orientation and race, ethnicity and racism.

Reporting on sex and gender	N/A
Reporting on race, ethnicity, or other socially relevant groupings	N/A
Population characteristics	N/A
Recruitment	N/A
Ethics oversight	N/A

Note that full information on the approval of the study protocol must also be provided in the manuscript.

Field-specific reporting

Please select the one below that is the best fit for your research. If you are not sure, read the appropriate sections before making your selection.

☐ Life sciences ☐ Behavioural & social sciences ☒ Ecological, evolutionary & environmental sciences

For a reference copy of the document with all sections, see nature.com/documents/nr-reporting-summary-flat.pdf

Ecological, evolutionary & environmental sciences study design

All studies must disclose on these points even when the disclosure is negative.

Study description	The study characterized organic molecules in abyssal hydrothermal vent chimney samples using a metabolomics-inspired mass spectrometry strategy. The study design compared samples across different vent fields (Longqi, Kairei, Edmond) and vent activity levels (active vs. inactive). The analysis involved hierarchical clustering of mass spectral data to build a molecular-relatedness tree and trace geochemical transformation pathways.
Research sample	The samples are hydrothermal chimney fragments collected from three vent fields (Longqi, Kairei, and Edmond) along the Indian Ridge during the TS10 cruise (2018-2019) using the HOV Shenhai Yongshi. Sampling targeted chalcopyrite-rich inner conduit material from both active and inactive chimneys. These samples represent organic matter subjected to high-temperature hydrothermal alteration in ultraslow-spreading ridge environments.
Sampling strategy	Sampling was conducted using the manipulator arms of the HOV Shenhai Yongshi to collect chimney fragments. Sample sizes were determined by availability of chimney fragments and field constraints during the cruise; no statistical sample-size calculation was required as the study focuses on molecular pattern comparison rather than population inference. The selected samples were deemed sufficient as the strategy ensured broad coverage of different vent types (active/inactive), locations, and geochemical regimes, which was adequate to test for overarching natural trends rather than perform spatial statistical comparisons.
Data collection	Geochemical data were collected in the laboratory. Organic extracts were analyzed using GC–MS (EI mode) and FT–MS (–ESI mode). Data acquisition and deconvolution were performed using open-sourced platform madial. Mass spectra matching etc. were processed using automated deconvolution and similarity-based annotation routines.
Timing and spatial scale	Laboratory analyses and molecular similarity computation were performed and collected in spring Jan to April 2025.
Data exclusions	No data were excluded.
Reproducibility	Following the established best practices in organic geochemistry, reproducibility was ensured during method establishment by running standards and blanks repeatedly until the signal was stable and baseline is clean, establishing stable background and repeatable signal profiles. Specifically, the reproducibility and repeatability of FT-MS results were systematically evaluated through replicates (quality control samples were analyzed multiple times within the same session and across different operators and time periods, showing stable results with low relative standard deviations, including both intra-day and inter-day evaluations), ensuring the robustness and consistency of the data across time and operators. During co-annotation and similarity analysis, to cross-validate reproducibility, spectral similarity thresholds were applied at three levels (20%, 50%, 70%) and only compounds with >70% similarity were retained for final interpretation. This cross-validation confirmed that the key molecular groupings and the overall topology of our phylogenetic tree were stable and not artifacts of a specific parameter choice. This approach ensures that the patterns we report are statistically sound and reproducible from the metadata. For both Hg concentrations and isotopes, replicate measurements of international standard reference materials yielded recoveries and relative standard deviations. Method blanks were consistently below detection limit, ensuring that no contamination was introduced during sample preparation. Replicate analyses of the same sample were carried out until stable values were obtained, and uncertainties are reported as 2SD based on repeated measurements of standards and samples.
Randomization	Not relevant to our study. Sample allocation was determined by vent activity (active vs inactive) and geological setting rather than random assignment, as the study investigates natural environmental gradients. Samples were categorized based on their pre-existing, natural geological attributes: their specific vent field of origin (Longqi, Kairei, Edmond) and their observed hydrothermal

	activity level (active vs. inactive).
Blinding	Not relevant to our study. Data were acquired using analytical instrumentation (GC-MS, FT-ICR-MS) and processed via a deterministic computational workflow .

Did the study involve field work? ☐ Yes ☒ No

Reporting for specific materials, systems and methods

We require information from authors about some types of materials, experimental systems and methods used in many studies. Here, indicate whether each material, system or method listed is relevant to your study. If you are not sure if a list item applies to your research, read the appropriate section before selecting a response.

Materials & experimental systems

n/a	Involved in the study
☒	☐ Antibodies
☒	☐ Eukaryotic cell lines
☒	☐ Palaeontology and archaeology
☒	☐ Animals and other organisms
☒	☐ Clinical data
☒	☐ Dual use research of concern
☒	☐ Plants

Methods

n/a	Involved in the study
☒	☐ ChIP-seq
☒	☐ Flow cytometry
☒	☐ MRI-based neuroimaging

Plants

Seed stocks	N/A
Novel plant genotypes	N/A
Authentication	N/A
